# Network Pharmacology-Based Investigation of Protective Mechanism of *Aster tataricus* on Lipopolysaccharide-Induced Acute Lung Injury

**DOI:** 10.3390/ijms20030543

**Published:** 2019-01-28

**Authors:** Yijun Chen, Jiaojiao Dong, Jie Liu, Wenjuan Xu, Ziyi Wei, Yueting Li, Hao Wu, Hongbin Xiao

**Affiliations:** Research Center of Chinese Medicine Analysis and Transformation & School of Chinese Materia Medica, Beijing University of Chinese Medicine, Beijing 100029, China; 18810619033@163.com (Y.C.); djjllzx@163.com (J.D.); znyspa.xf@163.com (J.L.); cathy_xwj@126.com (W.X.); zywei94@163.com (Z.W.); yuetingli1111@163.com (Y.L.); wuhao920817@163.com (H.W.)

**Keywords:** acute lung injury, *Aster tataricus*, inflammatory cytokines, vascular endothelial

## Abstract

Acute lung injury (ALI) is a common clinical condition that badly influences people’s health. Recent studies indicated that *Aster tataricus* (RA) had potential effects on ALI, but the effective components and their mechanism is not clear. In this study, we found that the Fraction-75 eluted from RA extract could significantly protect the lipopolysaccharide (LPS)-induced ALI in mice, including alleviating the severity of lung pathology, attenuating the pulmonary edema, and reducing the release of inflammatory cells. Further ingredient analyses demonstrated that there were mainly 16 components in it, among which 10 components were collected according to their relative peak area and oral bioavailability. Next, the components-disease targets network suggested that the candidate components had extensive associations with 49 known therapeutic targets of ALI, among which 31 targets could be regulated by more than one component. Herein, GO functional and pathway analysis revealed that the common targets were associated with four biological processes, including the inflammatory response to stimulus, cellular process, chemokine biosynthetic process and immune system process. Furthermore, the ELISA validation indicated that the candidate components in RA extract may protect the LPS-induced ALI mainly through inhibiting the release of inflammatory cytokines and promoting the repair of vascular endothelial.

## 1. Introduction

Acute lung injury (ALI) is characterized by uncontrolled progressive lung inflammation and it can further develop into its severe stage, acute respiratory distress syndrome (ARDS) [1,2]. As it was reported, ALI and ARDS can lead to acute respiratory failure with a high mortality rate of 40–60% and they may result from various pathologies, such as microbial infection, sepsis, trauma, reperfusion, etc. [3,4]. Although, advanced investigations have been made to improve the understanding of the pathophysiology of ALI and ARDS, there is still a lack of effective therapeutic strategy and drugs for treating either. Various molecular mechanisms are associated with the pathogenesis and progression of ALI, including inflammatory responses, oxidative stress, vascular endothelial cell proliferation, migration, apoptotic, immune system process, etc. Meanwhile, ALI and ARDS also have the characteristic of excessive neutrophil and macrophage infiltration into the lung tissues, release of pro-inflammatory cytokines (such as interleukin (IL)-1β, IL-6, and tumor necrosis factor (TNF)-α) and lung endothelial and epithelial injuries as well, resulting in edema and gas exchange deterioration [5,6,7,8]. 

The root of *Aster tataricus* (RA) is one of the most commonly used traditional Chinese herb, recorded in all versions of Chinese pharmacopoeia, with the functions of moistening lung, eliminating phlegm and relieving cough for more than two thousand years in China [9,10]. Modern pharmacology researches demonstrated that the extract of RA has extensive pharmacological activities, for example RA could effectively manages the diabetic retinopathy by controlling the blood glucose [11], relieve neuroinflammation by decreasing oxidative stress and attenuates the cytokines [12], etc. Meanwhile, researches also showed that RA and some compounds isolated from it, such as 4-hydroxyphenylacetic acid and shionone exhibited potential therapeutic effects in treatment of lung injury [13,14]. Although, lots of researches have been carried out to study on RA, there are still several scientific questions like the main bioactive components associated with efficacy and the interaction between these compounds in treatment of ALI that need to be solved.

The efficacy of TCM (traditional Chinese medicine) usually depends on the comprehensive effects of its components. Therefore, the study of the efficacy of TCM from the perspective of pharmacology alone cannot fully explain its scientific connotation [15]. Fortunately, with the rapid development of bioinformatics, network pharmacology, an emerging discipline based on the basic theories of systems biology and polypharmacology, has gradually become a powerful tool to investigate medicinal herbs action and mechanisms by mapping drug-target-disease networks from the biological level [16,17,18,19]. Meanwhile, as it was reported, lipopolysaccharide (LPS) is an endotoxin released from dead Gram-negative bacteria, which could cause leukocytosis, diffuse intravascular coagulation and endotoxic shock [20]. And LPS is also believed to have an important impact on the inflammatory response of ALI and widely used to establish ALI models [21]. Further investigations showed that the symptoms raised by LPS in animals, are very similar to that of human ALI [22].

**Figure 1:**
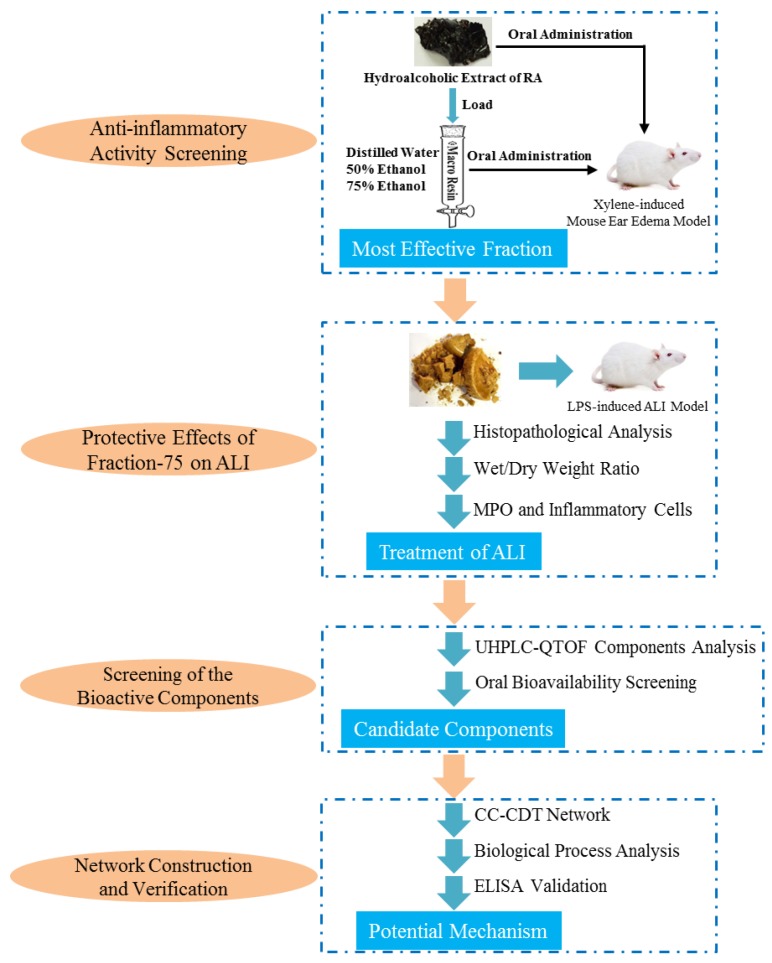
A flowchart to schematically describe the experimental procedure in this study.

As it was shown in Figure 1, in this present study, the xylene-induced mouse ear edema model was applied to preliminary screen out the anti-inflammatory fraction of RA extract. On this basis, the LPS-induced ALI model was then carried out to further evaluate its protective effects. After that, the major constituents in it were subsequently identified and candidate components were selected for further network pharmacology investigations. This work provides valuable methodological guidance to visualizing the interaction of drug and disease, which is beneficial for better understanding the complex mechanisms of multi-target therapy.

## 2. Results

### 2.1. Anti-Inflammatory Activity of RA and its Eluted Fractions

**Figure 2:**
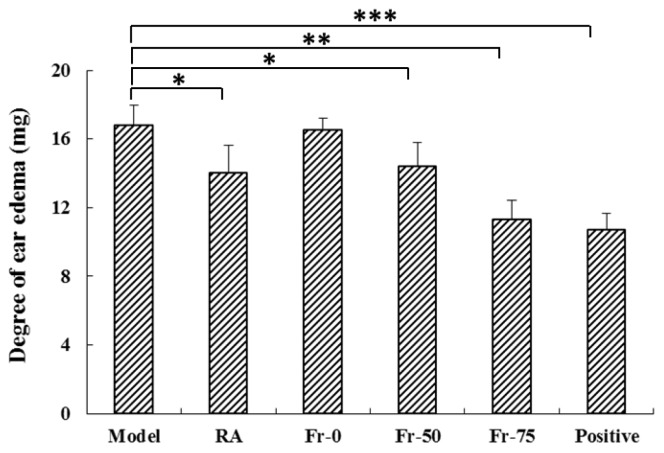
Effects of *Aster tataricus* (RA) and its three eluted fractions on xylene-induced ear edema. Mice were separately treated with RA extract (3.5 g/kg), Fr-0 (80 mg/kg), Fr-50 (80 mg/kg) and Fr-75 (80 mg/kg). Dexamethasone acetate (25 mg/kg) was considered as a positive control. Results are mean ± SE (*n* = 6), * *p* < 0.05, ** *p* < 0.01 and *** *p* < 0.001 compared with model group.

Xylene-induced mouse ear swelling model is usually used in the preliminary screening of anti-inflammatory drugs [23]. As it was shown in Figure 2, the average ear weight gain for the model, RA, Fraction-50, Fraction-75 and positive group was 16.8, 14, 14.4, 11.3, and 10.7 mg respectively. RA extract, at the dose of 3.5 g/kg, could significantly decrease the mouse ear edema by 16.7% (*p* < 0.05). And treatment with Fraction-50 and Fraction-75, at the dose of 80 mg/kg, could separately distinctly inhibited the ear edema by 14.3% (*p* < 0.05) and 26.8% (*p* < 0.05). Interestingly, the anti-inflammatory activity of Fraction-75 was almost the same as that of Dexamethasone acetate group (25.6%, 25 mg/kg). 

### 2.2. Protective Effects of Fraction-75 on LPS-Induced ALI

#### 2.2.1. Histopathological Changes, MPO Level, and W/D Weight Ratio in Lung Tissues

**Figure 3:**
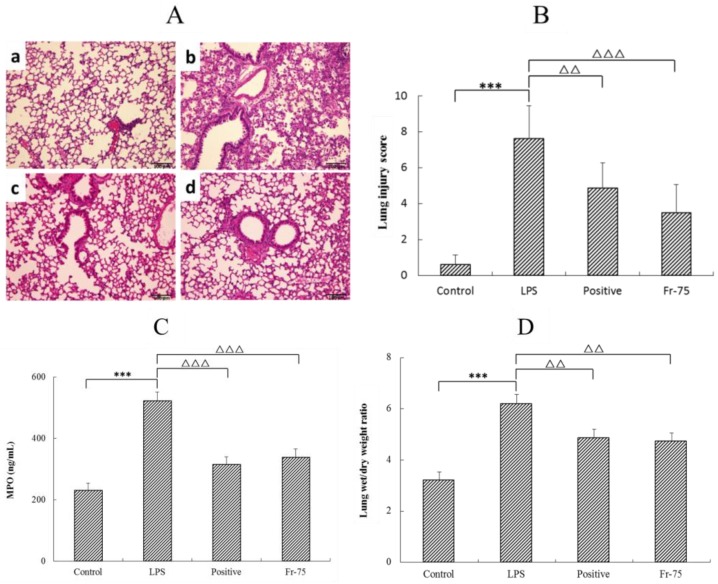
(**A**) Histopathological analysis of lung tissues, Hematoxylin and Erosin (H&E), original magnification ×200, scale bars 100 μm: (**a**) control group; (**b**) model group; (**c**) positive group; (**d**) Fraction-75 group; (**B**) Lung injury blind scoring of each group; (**C**) Concentration of myeloperoxidase of lung tissues in mice; (**D**) Lung wet/dry weight ratio of each group. Results are mean ± SE (*n* = 6), *** *p* < 0.001, compared with the control group and ^ΔΔ^ *p* < 0.01, ^ΔΔΔ^ *p* < 0.001, compared with the model group (lipopolysaccharide (LPS).

According to the anti-inflammatory screening results above, the Fraction-75 was collected to further evaluate whether it could also relieve the lung inflammation infected by LPS. As it was shown in Figure 3A, the control group (a) exhibited normal tissue structures with no histopathological changes. While, the LPS group (b) showed serious lung injury, expressing as full of inflammatory cell infiltration, edema, alveolar collapse, pulmonary interstitial hyperaemia, and haemorrhage. Gratifyingly, after oral administration of Fraction-75 (d), the degree of lung pathology injury was obviously reduced, manifested by the recovery of lung tissue structure and the decrease of inflammatory cells. Meanwhile, the Hematoxylin and Erosin (H&E) staining results also suggested that the efficacy of Fraction-75 group (d) was even better than that of the dexamethasone acetate (positive) group (c). Furthermore, the blind scoring results in Figure 3B were consistent with the pathological section results.

In addition, the level of myeloperoxidase (MPO) activity in lung tissue and the lung wet/dry (W/D) weight ratio were determined to confirm its efficacy on LPS-induced ALI. The results showed that in comparison with the control group, LPS could dramatically increase the MPO activity, which was significantly reduced with Fraction-75 treatment (Figure 3C). Coincidently, the lung tissues W/D weight ratio of LPS group is significant higher than that of control group. While, after administration of Fraction-75, the W/D weight ratio of lung tissues is distinctly decreased, indicating that Fraction-75 could effectively relieve the LPS-induced pulmonary edema (Figure 3D).

#### 2.2.2. Content of Inflammatory Cells in BALF

**Figure 4:**
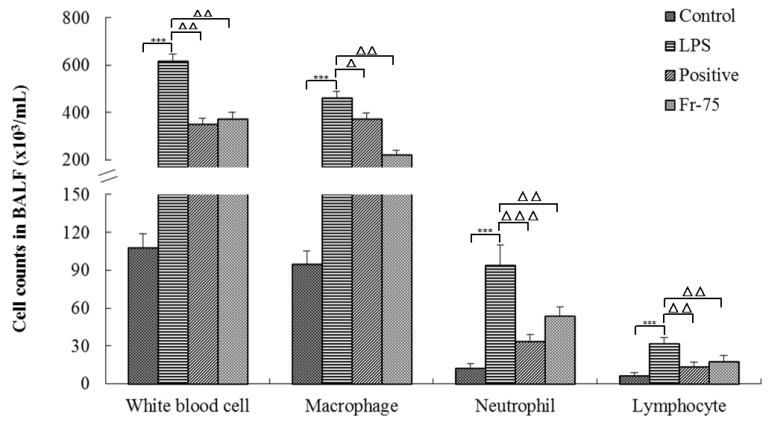
Counts of inflammatory cells in Bronchoalveolar Lavage Fluid (BALF). Results are mean ± SE (*n* = 6), *** *p* < 0.001, compared with the control group and ^Δ^ *p* < 0.05, ^ΔΔ^ *p* < 0.01, ^ΔΔΔ^ *p* < 0.001, compared with the model group (LPS).

Besides, the content of inflammatory cells in Bronchoalveolar Lavage Fluid (BALF) was also determined to evaluate its protective effects on LPS-induced ALI. As it was shown in Figure 4, LPS could significantly increase the counts of inflammatory cells in BALF compared with that of the control group. While, after administration of Fraction-75, the inflammatory cell counts were distinctly reduced suggesting that Fraction-75 may attenuate the lung inflammation through inhibiting the release of inflammatory cells.

### 2.3. Identification of the Candidate Components

#### 2.3.1. The Major Constituents of Fraction-75

**Figure 5:**
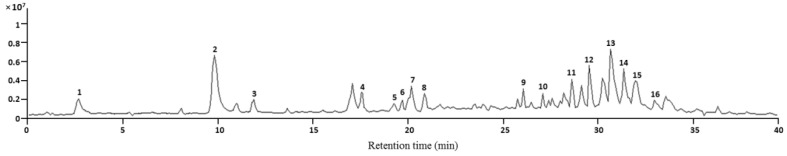
UHPLC-Q TOF total ion current (TIC) chromatogram of Fraction-75 in negative mode.

The UHPLC-Q TOF MS was employed to determine the constituents of Fraction-75. The total ion current (TIC) chromatogram of Fraction-75 in negative ion mode was listed in Figure 5. Based on the retention time, UV absorption spectra, accurate mass and MS/MS fragments information, a total of 16 compounds (Table 1) were characterized in comparison with known compounds and consulting with literatures. Meanwhile, the relative content of assigned compounds was evaluated according to the TIC chromatogram and the relative peak area (RPA) of each component was figured as below: area of each peak/total area of peaks on TIC chromatogram × 100%. The identified compounds in Fraction-75 accounted for about 87.82% in total. Meanwhile, the chromatograms of RA extract and other fractions were provided in the supplementary material.

#### 2.3.2. Acquisition of the Candidate Components

On the basis of the components identification above, the traditional Chinese medicine systems pharmacology (TCMSP) database was used to collect the candidate components of RA. As it was shown in Table 2, 10 candidate components were obtained. And the standard of screening was as follows: oral bioavailability (OB) ≥20% and relative peak area (RPA) ≥2%. These 10 candidate components accounted for almost 70% of the total content. And they will be used in subsequent network pharmacological studies.

### 2.4. Construction of the Network and ELISA Verification of the Key Targets

#### 2.4.1. Candidate Components-Disease Targets Network

**Figure 6:**
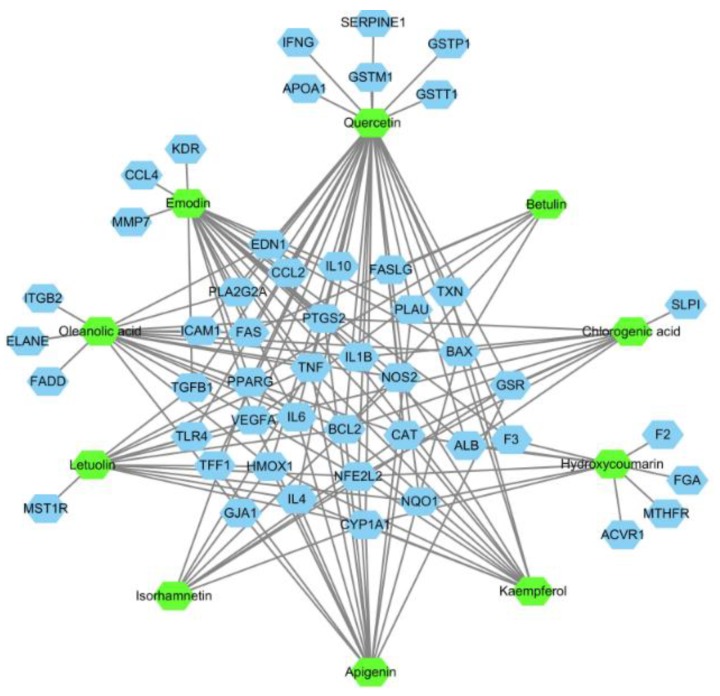
Components-disease targets (CC-DT) network of the 10 candidate components and the therapeutic targets in treatment of acute lung injury (ALI). The green nodes represent the candidate components and the blue nodes are disease targets.

Firstly, with the help of GisGeNET database (http://www.disgenet.org/), the known therapeutic targets of drugs in the treatment of ALI were collected. Then the targets with a relevance score ≥0.001 were retained and a total of 75 targets were obtained. Next, the GeneCard database was subsequently used to separately gather these 10 candidate components’ targets. After that the targets, which associated with both ALI and the candidate components, were screened out and regarded as disease targets for candidate components-disease targets (CC-DT) network construction. As it was shown in Figure 6, there were 10 components and 49 targets included in the CC-DT network. It could be found that the interactions with components and targets varies a lot, some candidate targets were hit by only one candidate component, while others could be modulated by multiple components. Among them, 31 targets associated with more than one component. And we will pay more attentions to these targets in our following research.

#### 2.4.2. Biological Process of the Components’ Targets

GO functional analysis and KEGG pathway analysis were then employed to further investigate the biological process of the component’s targets using Cytoscape software (version 3.6.1) based on the STRING score values (higher than 0.70 with high confidence). The targets in each candidate components were analyzed, respectively, removing the targets without the interaction of others. And the STRING networks were provided in the supplementary material. As it was shown in Table 3, the biological process analysis revealed that these components participated in several important metabolic processes in the organisms giving rise to a prospective therapeutic effect. Additionally, four common biological processes were discovered in these components, including the inflammatory response to stimulus, cellular process, chemokine biosynthetic process and immune system process, which constitute the main biological process of these components. Among all these targets, the IL-1β, TNF, and IL-6 were closely related to the regulation of the inflammatory cytokines release and chemokines biosynthetic and the vascular endothelial growth factor A (VEGFA) was mainly associated with the vascular endothelial cell migration, proliferation. 

#### 2.4.3. ELISA of the Key Targets

**Figure 7:**
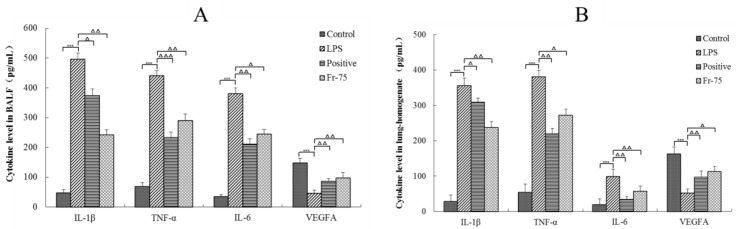
Concentrations of four cytokines in BALF (**A**) and in lung-homogenate (**B**). Results are mean ± SE (*n* = 6), *** *p* < 0.001, compared with the control group and ^Δ^ *p* < 0.05, ^ΔΔ^ *p* < 0.01, ^ΔΔΔ^ *p* < 0.001, compared with the model group (LPS).

The content of IL-1β, TNF-α, IL-6, and VEGFA in BALF and lung-homogenate were then determined to validate the prediction results of the network pharmacological investigations. As it was shown in Figure 7, the concentration level of IL-1β, TNF-α, and IL-6 in LPS group were obviously higher than those in control group (*p* < 0.001). Fortunately, the Fraction-75 could distinctly reduce the content of these cytokines in both BALF and lung-homogenate (*p* < 0.05). And the level of IL-1β in Fraction-75 group was even lower than that in positive group. Moreover, it could be also found that the LPS could significantly decrease the content of VEGFA in LPS group compared with control group (*p* < 0.001). While, after oral administration of Fraciont-75 the level of VEGFA was obviously increased. All these results were consistent with the prediction results of biological process analysis, suggesting that the active components in Fraction-75 may alleviate ALI by inhibiting the release of inflammatory cytokines and promoting the repair of vascular endothelial.

## 3. Discussion

The core theory of ALI is that the imbalance of inflammatory response aggravates the injury of epithelium or endothelium [24,25], resulting in the protein-rich fluid to enter the alveoli [26]. Increased permeability of alveolar epithelial microvessels in the process of ALI will eventually lead to ARDS [27]. Histologically, ALI is characterized by severe acute inflammation, massive apoptosis of alveolar epithelial, increased alveolar-capillary permeability, and formation of fibrosis [28]. The cellular pathology of ALI includes destruction of alveolar-capillary membrane integrity, excessive neutrophil migration and the promotion of inflammatory cytokines [29,30].

It is well known that macrophages and neutrophils are the primary source of diversified inflammatory mediators during the progression of ALI. They infiltrate into lung tissues and release enzymes and phagocytize pathogens, and they are a fundamental source of inflammatory regulators in vivo. In the early stage of LPS-induced ALI, neutrophils attach and accumulate in the pulmonary capillaries, then migrate to the alveolar lumen, where they are activated, and release cytotoxic substances (oxygen radicals, lipid mediators, and proteases), leading to damage of alveolar epithelial cells and capillary endothelial cells [31,32]. Alveolar macrophages are formed when monocytes migrate to lung tissues, and mainly distributed in the alveolar cavity with the functions of phagocytosis and secretion. Alveolar macrophages are critical to maintaining tissue balance and increasing rapid response to exogenous and endogenous stimuli in inflammatory pulmonary disease [33]. Alveolar macrophages are also the major source of various inflammatory cytokines, such as TNF-α, IL-1β, and IL-6, which play a critical role in inflammation [34]. Meanwhile, there is accumulating evidence indicating that lymphocytes have also played important roles in the development and progression of ALI. It has been suggested that lymphocytes contribute to the progression of autoimmune and inflammatory diseases [35]. These cells levels could be indicators supporting the role of LPS on ALI, and Fraction-75 could reduce 39.2% of white blood cells, 51.8% of macrophages, 63.8% of neutrophils, and 43.6% of lymphocytes in BALF compared with that of LPS group and thus achieve anti-inflammatory effects on ALI. Meanwhile, myeloperoxidase (MPO), which is mainly released by activated neutrophils, is a powerful pro-oxidative and pro-inflammatory enzyme and it could contribute to exacerbation and prolongation of inflammation. The concentration and activity of MPO in lung tissue homogenates usually considered as a surrogate marker of neutrophil infiltration [36,37]. The present study demonstrated that Fraction-75 could protect the ALI as well by reducing the expression of MPO.

Tumor necrosis factor (TNF)-α is a major pro-inflammatory and immunomodulatory factor in organisms and is involved in mediating the process of ALI. TNF-α can promote the expression of vascular endothelial cell adhesion molecules, stimulate the activation and migration of pulmonary endothelial cells and macrophages, and induce secretion of cytokines to trigger inflammatory responses [38,39]. And it has been reported that vasodilator stimulated phosphoprotein (VASP) is negatively regulated by TNF-α, which induces hypoxia-inducible factor-1α (HIF-1α) activation and plays an important role in the damage of alveolar capillary barrier [40]. Interleukin (IL)-1β is a classical innate immune cytokine which exists in the whole process of inflammation from the beginning to the end. It can effectively reflect the intrinsic inflammation of the respiratory tract and the amount of IL-1β will be increased in ALI and asthma [41,42]. IL-6 is mainly produced by the innate immune system and is one of the first cytokines released in the acute phase of ALI and is followed by increase in the expression of IL-1β, IL-8, TNF-α, and IL-10. IL-6 maintains tissue homeostasis and reflects the extent of tissue damage, which is critical in the inflammatory response [43]. In our study, the IL-1β, TNF-α, and IL-6 levels in BALF and lung tissues were evidently lower in the Fraction-75 group than in the LPS group, suggesting that the Fraction-75 may alleviate ALI by reducing the concentrations of pro-inflammatory cytokines. In addition, vascular endothelial growth factor A (VEGFA) is growth factor active in angiogenesis and endothelial cell growth. It could induce endothelial cell proliferation, promote cell migration, inhibit apoptosis and improve permeabilization of blood vessels. VEGFA significantly reduces the lung structural damage and neutrophil infiltration induced by LPS in convalescence mice [44]. Fortunately, Fraciont-75 could significantly increase the level of VEGFA in comparison with the LPS group.

**Figure 8:**
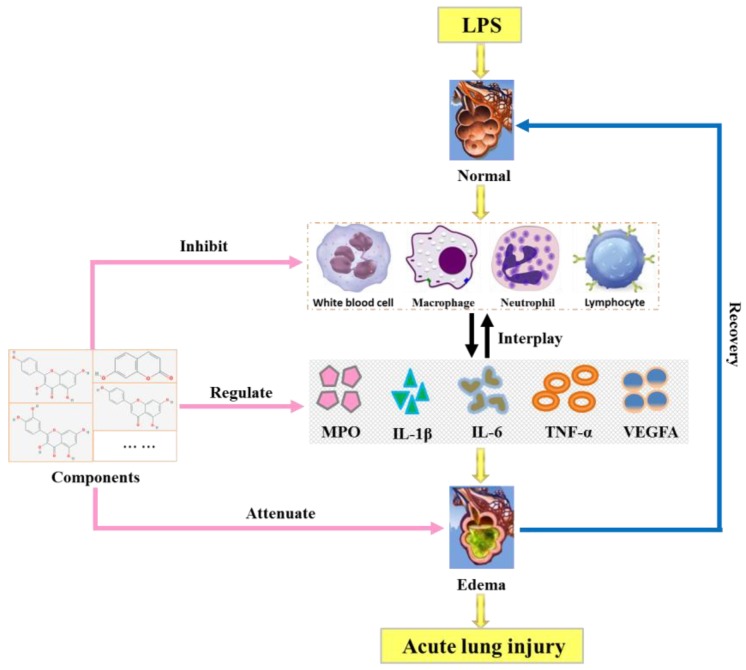
Mechanism of the candidate components in the treatment of LPS-induced ALI.

As we all known, the identification of network targets is one of the core issues used to reveal the molecular mechanism of traditional Chinese medicine (TCM). The network analysis based on widely existing databases could help people to form a better understanding of the mechanism by which TCM works [45]. In our present study, the candidate components-disease targets (CC-DT) network was constructed and the biological process of drug effect of candidate components in RA was revealed and visualized with the aid of the CC-DT and STRING network. Ten candidate components were involved in the CC-DT network, and four biological processes were included in the STRING network. Four key targets, which closely related to two biological processes (inflammatory response to stimulus and cellular process) in ALI, were directly linked to both the components and disease. Meanwhile, according to the retrieval results of database and literature reports, the flavonoids (quercetin, kaempferol, apigenin, luteolin, and isorhamnetin) in CC-DT network have extensive pharmacological activities [46,47,48,49] on LPS-induced ALI, especially in anti-inflammation and vascular repair. By the way, the chlorogenic acid, oleanolic acid [50], botulin [51], and emodin had also been reported about their anti-inflammatory activities: chlorogenic acid could attenuate the LPS-induced mice mastitis by inhibiting the TLR4-mediated NF-κB pathway [52], emodin could relieve NLRP3 inflammasome activation, leading to decreased secretion of IL-1β and blocking of the inflammasome-induced pyroptosis [53], indicating that they may take responsibility for the anti-inflammatory activities as well. Generally, as it was shown in Figure 8, candidate components in RA extract may protect the LPS-induced ALI mainly through the following ways: inhibiting the release of inflammatory cells, regulating of the pro-inflammatory cytokines, attenuating the pulmonary edema, etc.

## 4. Materials and Methods

### 4.1. Reagents and Chemicals

Chloral hydrate, xylene and Wright-Giemsa stain were purchased from Nanjing Chemical Reagent Co. (Nanjing, China). HPD-100 macroporous resin was purchased from Tianjin Haiguang Chemical Co., Ltd. (Tianjin, China). Dexamethasone acetate, lipopolysaccharide (LPS), and other compounds with the purity >95% were purchased from the National Institute for Food and Drug Control (Beijing, China). HPLC-grade acetonitrile, methanol and formic acid were supplied by Merck (Darmstadt, Germany). BCA protein assay kit, mouse myeloperoxidase (MPO), TNF-α, IL-1β, IL-6 and VEGFA ELISA kit were purchased from Proteintech Group Inc. (Chicago, IL, USA).

### 4.2. Sample Preparation

RA was purchased from Beijing Tongrentang and identified by Professor Xueyong Wang. The experimental specimens were placed in the laboratory of Beijing University of Chinese Medicine (No.CMAT-AT-201604). RA (1 kg) was crushed and extracted by the following process: 75% ethanol (*v/v*), 1:10 of solid-liquid ratio, and 2 h of each reflux extraction for two times. Then, the resulting solution was filtered and concentrated. The concentrated extract named RA extract. Then, the RA extract was loaded onto a column (120 × 10 cm) of D-101 macroporous resin. After that, 3-bed column of water, 50% and 75% ethanol (*v/v*) were sequentially poured into the column with a flow rate of 10 mL/min to separately get three eluted fractions, named Fraction-0, Fraction-50 and Fraction-75. After freeze-drying, the yield of RA extract, Fraction-0, Fraction-50 and Fraction-75 are 30.5, 11.3, 6.5 and 7.2% respectively. 

### 4.3. Animals

KM mice (18–22 g) with half males and half females were purchased from Beijing Vital River Laboratory Animal Technology Co., Ltd. Water and foods were plentiful and free to the animals. Meanwhile, the room temperature (25 ± 2 °C) and humidity (50 ± 5%) were stable coupled with a 12 h light/dark cycle. This study was carried out in accordance with the recommendations of the Principles of Laboratory Animals and the related ethical regulations of Beijing University of Chinese Medicine. The protocol was approved by the ‘Affidavit of Approval of Animal Ethical and Welfare of BUCM’ (No.4-2017010103-10043, 27 September 2017, Beijing University of Chinese Medicine).

### 4.4. Establishment of Mouse Ear Edema Model and Screening of the Effective Fraction

The xylene-induced mouse ear swelling model was carried out in the first place to preliminary evaluate the anti-inflammatory activity of RA extract and its eluted fraction. Mice were randomly divided into six groups (*n* = 6) and separately treated with saline (control), RA extract (3.5 g/kg), Fraction-0 (80 mg/kg), Fraction-50 (80 mg/kg), Fraction-75 (80 mg/kg) and dexamethasone acetate (positive, 25 mg/kg) once daily for 3 days in a row, and the dosage of each group was based on the principle of Chinese Pharmacopoeia. Thirty minutes later after the last administration, 0.02 mL of xylene was evenly smeared on both sides of the right ear of mice and nothing with the left ear. Waiting for another thirty minutes treatment mice were sacrificed through cervical dislocation and ears were carefully removed. Then the isolated ears were punched into ear disks (8 mm in diameter), and weighed. The degree of ear swelling was indicated by the weight of right ear minus the weight of the left ear (mg) and the inhibition rate of ear swelling was calculated using the formula below: Inhibition (%) = (Wc–Wt)/Wc × 100%, ‘Wc’ is represented for the average ear weight increase in control group and ‘Wt’ is represented for that in treatment group.

### 4.5. Set Up of Acute Lung Injury (ALI) Model

Similar to the method described in ‘4.4’, mice were randomly divided into four different groups, including control, LPS, positive and Fraction-75 group. Then a well-accepted method was employed to establish the ALI model [54]. Briefly, mice were intranasally instilled with 2 mg/kg LPS every 24 h for a total of 3 times, and the control group received equal volume of saline. After that, the treated groups received an oral administration with certain drugs and doses once daily for 5 days in a row, and the control group and LPS group received equal volumes of saline. Half of the mice got a tracheostomy and inserted with an endotracheal tube into their trachea under anesthesia. Later on, ice-cold PBS (total volume: 1.4 mL) was used to fill the lung two times to obtain the Bronchoalveolar lavage fluid (BALF). And the other half of the mice was used to collect the lung tissue.

### 4.6. Evaluation of the Histopathological and Wet/Dry Weight Ratio of Lung Tissues 

Part of the lung tissues were cut into sections of approximately 0.5 cm^2^ sizes. Then the segments of these lung tissues were fixed on a 4% paraformaldehyde solution for 48 h and embedded in paraffin. After that the samples were deparaffinized, hydrated and stained with hematoxylin and erosin (H&E) to assess the lung injury by pathological sectioning (*n* = 6 of each group). Neat, an investigator, who was initially blinded to the research groups, was employed to evaluate the histological images. The lung injury was scored according to the following principle: (1) alveolar congestion, (2) hemorrhage, (3) infiltration or aggregation of neutrophils in the airspace or vessel wall, and (4) thickness of the alveolar wall/hyaline membrane formation. Each item was scored on a 5-point scale as follows: no damage or minimal damage = 0; mild damage = 1; moderate damage = 2; severe damage = 3; diffuse injury = 4. All scores were added up to obtain a total score [55]. In addition, in order to further evaluate the pulmonary edema, the lung wet/dry (W/D) weight ratio was measured by dividing the wet weight by the dry weight. The lung tissues were removed and weighed (*n* = 6 of each group). Subsequently, they were placed in an incubator for 24 h at 80 °C to obtain the dry weight [56].

### 4.7. Measurement of the MPO and Inflammatory Cells

The level of MPO activity is often applied to predict the early risk of inflammatory diseases [57]. The lung tissues were homogenized with reaction buffer for MPO levels assay using an MPO commercial sandwich enzyme-linked immunosorbent assay (ELISA) kit according to the manufacturer’s instructions. Briefly, 100 μL of MPO standard solutions and 100 μL of samples were added to proper wells and incubated at 37 °C for 90 min. Then each well was washed with 100 μL of PBS 3 times. After that, avidin-biotin-peroxidase complex working solution was added to each well and incubated at 37 °C for another 30 min. Finally, 90 μL of TMB color developing agent and 100 μL of TMB stop solution were added to each well and the absorbance was measured at 450 nm. 

The contents of the inflammatory cells (white blood cells, macrophages, neutrophils and lymphocytes) could help to further evaluate the therapeutic effects of Fraction-75 on LPS-induced ALI. The BALF was centrifuged at 2000 rpm for 10 min and the pellet was resuspended in 0.5 mL PBS. After that they were stained with Wright-Giemsa stain. Then, the inflammatory cells were counted under a microscope. 

### 4.8. UHPLC-QTOF MS Analysis of Fraction-75

The components analysis was as follows: Aglient 1290 UHPLC instrument with auto sampler (G4226A), diode array detector (G4212A), quaternary pump (G4220A), column compartment (G1316C) and mass spectra (6550, Agilent Technologies, Palo Alto, CA, USA). Samples were separated by ACQUITY UPLCR HSS T3 (100 mm × 2.1 mm, 1.8 μm, Waters, Milford, MA, USA), the temperature was 30 °C, the detection wavelength was 254 nm, and the mobile phase was made up of acetonitrile (A) and 0.1% formic acid (B), gradient elution (*v/v*): 0 min, 5% A; 5 min, 20% A; 12 min 35% A; 20 min 50% A; 35 min 80% A; and 40 min 95% A. The injection volume was 2 μL. The MS parameters were shown below: analysis was carried out in negative mode and the mass range was set at 100–1200 Da. Conditions of the ESI source: Drying Gas (N_2_), 10 L/min; Gas Temp, 230 °C; Nebulizer, 45 psig; Sheath Gas Flow, 12 L/min; Sheath Gas Temp, 300 °C; Capillary Voltage, 3500 V (negative mode). The sample collision energy was set at 10, 20 and 40 V. Data were processed by the MassHunter Workstation software (version B.07.00, Agilent Technologies, USA).

### 4.9. Investigations of the Network Pharmacology

On the basis of the components analysis results and their oral bioavailability in TCMSP database (http://lsp.nwu.edu.cn/tcmspsearch.php), the candidate components were obtained. The GeneCards database (https://www.genecards.org/) was then used to capture the candidate components targets. Meanwhile the DisGeNET database (http://www.disgenet.org/) was used to get the known therapeutic targets of drugs in the treatment of ALI. After removing the duplicate targets and deleting the targets with a relevance score <0.001, the therapeutic targets associated with ALI were obtained. Next, the VENNY^2.1^ (http://bioinfogp.cnb.csic.es/tools/venny/index.html) was further used to intersect the candidate components’ targets with the potential targets of ALI to reduce the number of targets for further network construction. On this basis, the Cytoscape software (version 3.6.1) was then employed to construct the candidate components-disease targets (CC-DT) network by linking the candidate components’ targets and the therapeutic targets of ALI. Finally, the common targets, which associated with more than one component, of each component were separately used to conduct the biological process analysis. 

### 4.10. Determination of the IL-1β, TNF-α, IL-6, and VEGFA

To verify the network pharmacology prediction results, four key cytokines (IL-1β, TNF-α, IL-6 and VEGFA) in BALF and lung-homogenate were subsequently determined. Similar to the method described in ‘4.7’, the ELISA kits of IL-1β, TNF-α and VEGFA were separately employed to measure the concentrations of these cytokines according to the manufacturer’s recommendations. To ensure the accuracy of the experiment, each group (*n* = 6) was measured three times in parallel to obtain the average value for data analysis.

### 4.11. Statistical Analysis

Statistical analysis was performed with GraphPad Prism 5 software program. Values were expressed as mean ± SE (standard error). The MPO, TNF-α, IL-1β, and VEGFA activities of each group were evaluated with ANOVA. Values of *p* < 0.05 were considered to the statistically significant.

## 5. Conclusions

In this study, the Fraction-75, eluted from RA extract, could not only significantly inhibit the xylene-induced mouse ear edema, but could also protect the LPS-induced ALI from multiple aspects, including alleviating the severity of lung pathology, attenuating the pulmonary edema, reducing the release of inflammatory cells. Further network pharmacology investigations showed that 10 candidate components included in RA extract could protect the LPS-induced ALI by inhibiting the release of inflammatory cytokines and promoting the repair of vascular endothelial. This work provided a practical way to elucidate the relevance of the ingredients and their therapeutic effect, and to help people towards a better understanding of the biological process regulated by these ingredients.

## Figures and Tables

**Table 1:** Compounds identified by UHPLC-Q TOF in Fraction-75 of *A. tataricus* and their relative contents.

Peak No.	RT (min)	[M-H]^-^ (*m*/*z*)	Formula	Assignment	RPA ^a^ (%)	Error (ppm)	MS/MS Fragments (*m*/*z*)
1	2.63	301.0723	C_16_H_14_0_6_	Quercetin ^b^	4.43	−1.79	178.9970, 151.0021, 121.0283, 65.0024
2	9.82	285.0411	C_15_H_10_O_6_	Kaempferol ^b^	14.63	−2.24	255.0269, 227.0321, 183.0420, 117.0337
3	11.81	315.0514	C_16_H_12_O_7_	Isorhamnetin ^b^	4.03	−1.19	300.0246, 151.0021, 107.0124
4	17.33	285.0411	C_15_H_10_O_6_	Luteolin ^b^	3.41	−2.24	267.1959, 178.9063, 131.8974, 67.0191
5	19.12	353.0886	C_16_H_18_O_9_	5-Caffeoylquinic acid ^b^	1.89	−2.25	191.0559, 179.0352, 135.0448
6	19.56	353.0887	C_16_H_18_O_9_	4-Caffeoylquinic acid ^b^	1.73	−2.53	191.0560, 179.0352, 173.0450, 135.0444
7	20.02	353.0868	C_16_H_18_O_9_	Chlorogenic acid ^b^	5.85	2.57	191.0559, 85.0293
8	20.73	353.0872	C_16_H_18_O_9_	1-Caffeoylquinic acid ^b^	3.26	1.72	191.0566
9	25.92	269.0449	C_15_H_10_O_5_	Emodin ^b^	3.05	2.40	241.0122, 213.0183, 197.0226, 161.0273
10	25.93	431.0991	C_21_H_20_O_10_	Apigenin 7-glucoside	1.89	−1.69	277.2140, 171.0044, 152.9944, 96.9689
11	28.37	441.3748	C_30_H_50_O_2_	Betulin	5.03	−2.26	167.0002, 122.9745, 96.9591, 79.9567
12	29.29	269.0462	C_15_H_10_O_5_	Apigenin ^b^	7.94	−2.43	225.0510, 117.0331, 107.0121, 83.0123
13	30.42	161.0242	C_9_H_6_O_3_	hydroxycoumarin	12.81	1.35	117.0704, 91.0545, 62.0163
14	31.12	425.3795	C_30_H_50_O	Taraxerol ^b^	7.83	−1.43	392.2623, 211.0295, 174.8617, 96.9593
15	31.77	455.3542	C_30_H_48_O_3_	Oleanolic Acid	8.28	−2.48	407.1013, 391.4235, 377.2357, 363.0071
16	32.83	435.3126	C_26_H_44_O_5_	Terpene ^c^	1.76	−2.3	152.9946, 78.9585

^a^ Relative peak area; ^b^ Compared with authentic compounds; ^c^ (13S)-13-[(6-deoxy-alpha-l-mannopyranosyl)oxy]labda-8(20),14-dieneCompounds.

**Table 2:** Information of the 10 candidate compounds.

Compound	OB (%)	RPA (%)	CAS
Quercetin	46.43	4.43	117-39-5
Kaempferol	41.88	14.63	520-18-3
Isorhamnetin	49.60	3.03	480-19-3
Luteolin	36.16	3.41	491-70-3
Chlorogenic acid	24.50	5.85	327-97-9
Emodin	24.40	2.05	518-82-1
Betulin	20.48	4.03	473-98-3
Apigenin	23.06	7.94	520-36-5
Hydroxycoumarin	25.36	12.81	93-35-6
Oleanolic acid	29.02	8.28	508-02-1

**Table 3:** Biological process analysis of the candidate components’ targets.

Compound	Key Relevant Targets	Biological Process
Quercetin	IL6, IL1B, TNF, PTGS2	regulation of chemokine biosynthetic process (56.25%)
	TNF, IL10, VEGFA	regulation of chronic inflammatory response to antigenic stimulus (18.75%)
	BAX, VEGFA	positive regulation of B cell apoptotic process (12.5%)
	CCL2, ICAM1	negative regulation of vascular endothelial cell proliferation (9.38%)
	BAX	retinal cell programmed cell death (3.12%)
Kaempferol	PPARG, HMOX1, TNF	regulation of vascular smooth muscle cell proliferation (33.33%)
	CAT, CYP1A1, PPARG	response to hyperoxia (26.67%)
	IL4, NFE2L2, TNF	endothelial cell apoptotic process (20.0%)
	TXN, BAX, BCL2	homeostasis of number of cells within tissue (20.0%)
Isorhamnetin	TNF, NFE2L2	regulation of removal of superoxide radicals (40.0%)
	TNF, HMOX1	positive regulation of chemokine biosynthetic process (40.0%)
	HMOX1, NFE2L2	regulation of transcription from RNA polymerase II promoter in response to oxidative stress (20.0%)
Luteolin	TNF, IL4, IL1B, TGFB1, HMOX1	cytokine production involved in immune response (75.93%)
	TNF, IL6, HMOX1	regulation of chemokine biosynthetic process (18.52%)
	VEGFA, HMOX1, PPARG, TGFB1	regulation of blood vessel endothelial cell migration (3.7%)
	VEGFA, TGFB1, BCL2	branching involved in ureteric bud morphogenesis (1.85%)
Chlorogenic acid	IL1B, BCL2, BAX	programmed cell death involved in cell development (55.56%)
	ALB, CAT, GSR, NFE2L2	cellular oxidant detoxification (33.33%)
	VEGFA, PTGS2, NFE2L2	positive regulation of blood vessel endothelial cell migration (11.11%)
Emodin	TNF, IL1B, IL6, PTGS2	positive regulation of acute inflammatory response (68.09%)
	TNF, IL10, VEGFA	regulation of chronic inflammatory response to antigenic stimulus (19.15%)
	PPARG, TGFB1	negative regulation of vascular endothelial cell proliferation (12.77%)
Betulin	BCL2, BAX, FASLG	retinal cell programmed cell death (33.33%)
	VEGFA	monocyte differentiation (25.0%)
	VEGFA, BAX	post-embryonic camera-type eye development (16.67%)
	FAS, BAX	positive regulation of cysteine-type endopeptidase activity involved in apoptotic signaling pathway (16.67%)
	FASLG, FAS	necroptotic signaling pathway (8.33%)
Apigenin	TNF, IL6, HMOX1	regulation of chemokine biosynthetic process (42.86%)
	VEGFA, HMOX1	positive regulation of blood vessel endothelial cell proliferation involved in sprouting angiogenesis (14.29%)
	VEGFA	positive regulation of transcription from RNA polymerase II promoter in response to hypoxia (14.29%)
	BAX	retinal cell programmed cell death (14.29%)
	VEGFA, BAX	post-embryonic camera-type eye development (14.29%)
Hydroxycoumarin	NFE2L2	response to oxygen radical (41.67%)
	CYP1A1, CAT	response to hyperoxia (25.0%)
	F3	positive regulation of coagulation (25.0%)
	F3, CYP1A1	response to iron ion (8.33%)
Oleanolic acid	IL1B, TNF, IL6, HMOX1	regulation of chemokine biosynthetic process (83.33%)
	BAX, FAS, ICAM1	retinal cell programmed cell death (13.89%)
	CAT, PPARG	response to vitamin E (2.78%)

## Supplementary Materials

They are available online at http://www.mdpi.com/1422-0067/20/3/543/s1.
