# Supplementary material for: Network Pharmacology-Based Investigation of Protective Mechanism of Aster tataricus on Lipopolysaccharide-Induced Acute Lung Injury
Source: Int J Mol Sci. 2019 Jan 28;20(3):543. doi: 10.3390/ijms20030543 (PMC6387216; doi:10.3390/ijms20030543)

**(Figure 5) Supplementary material**

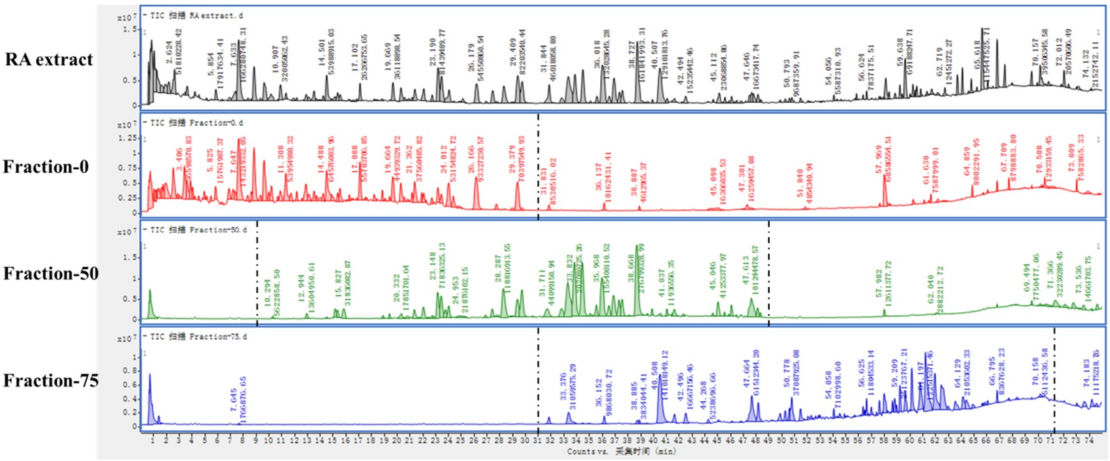


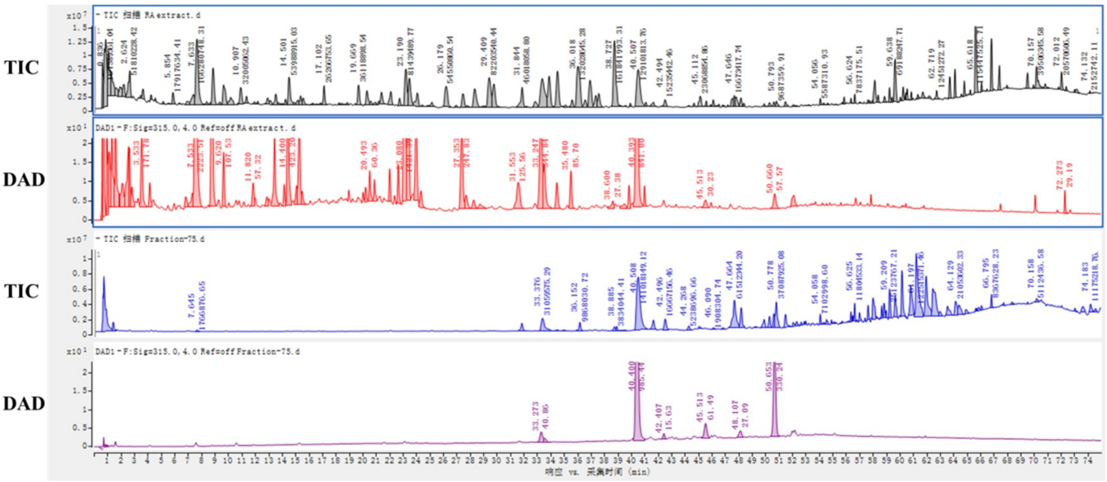

**(Table 3) Supplementary material**

Biological process analysis and the STRING network of the candidate components.

① Quercetin

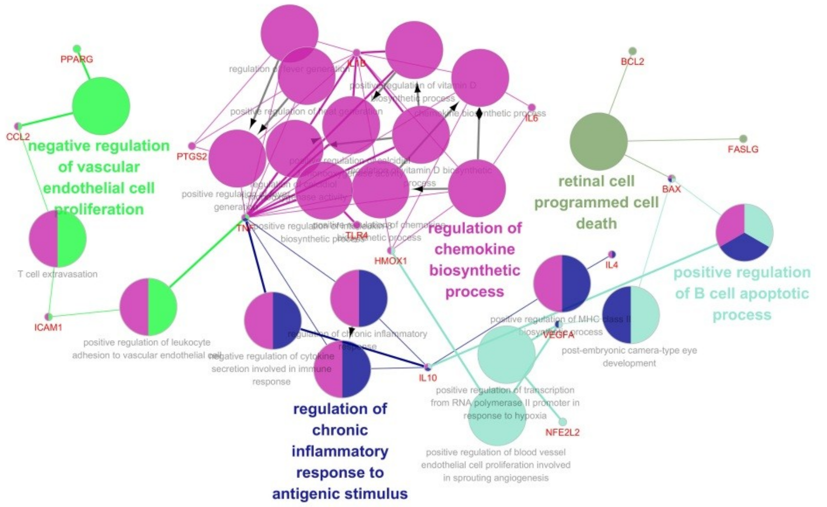


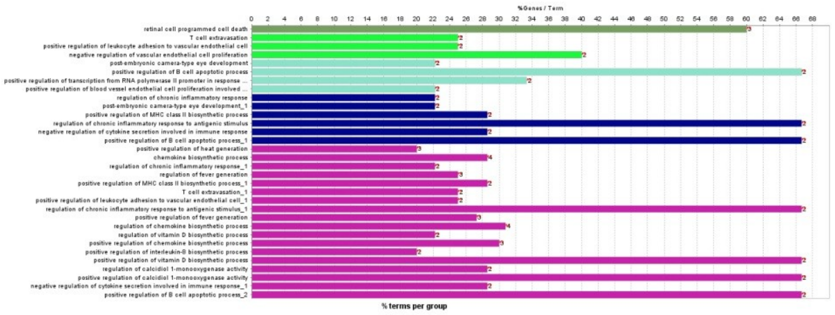


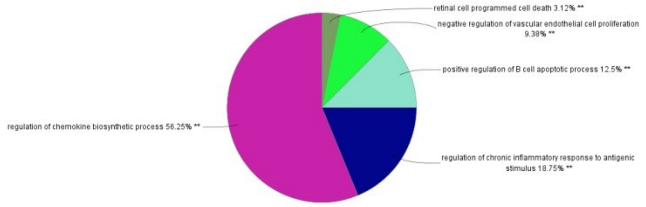


② Kaempferol

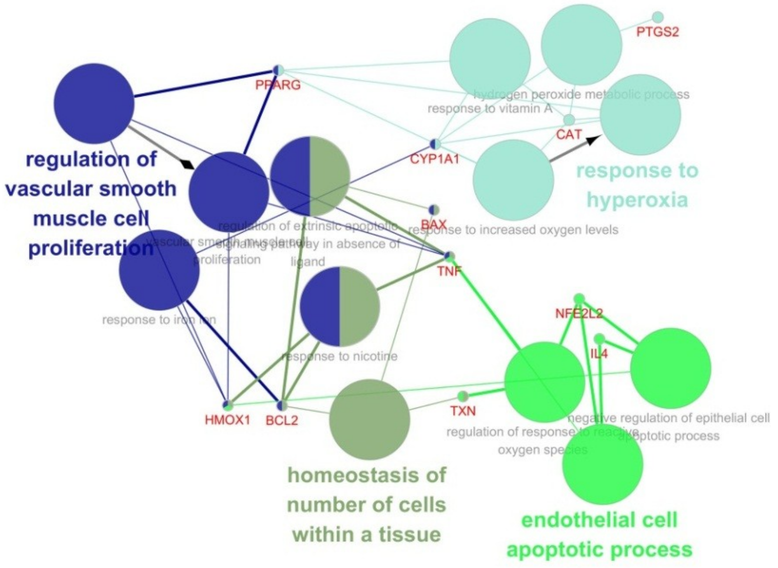

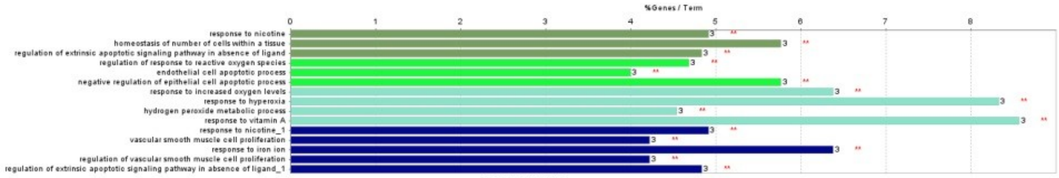


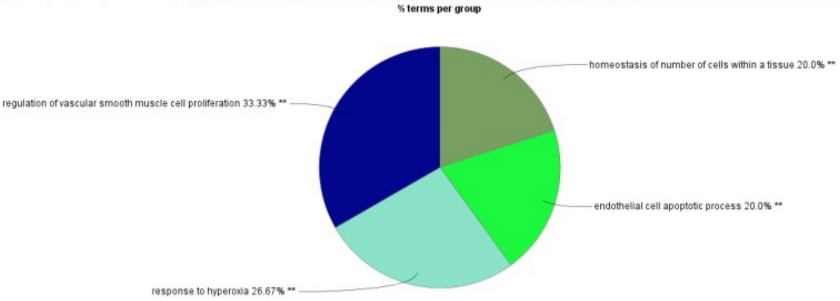


③ Isorhamnetin

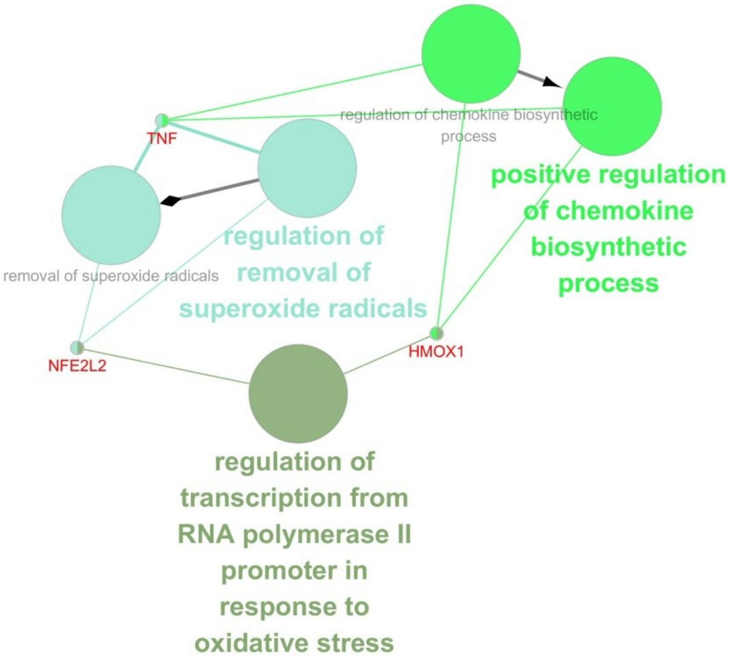


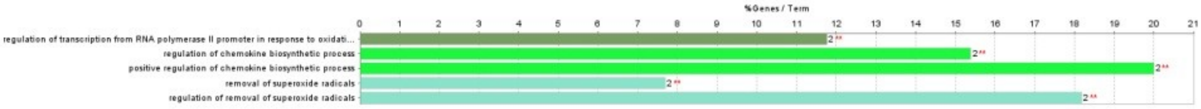


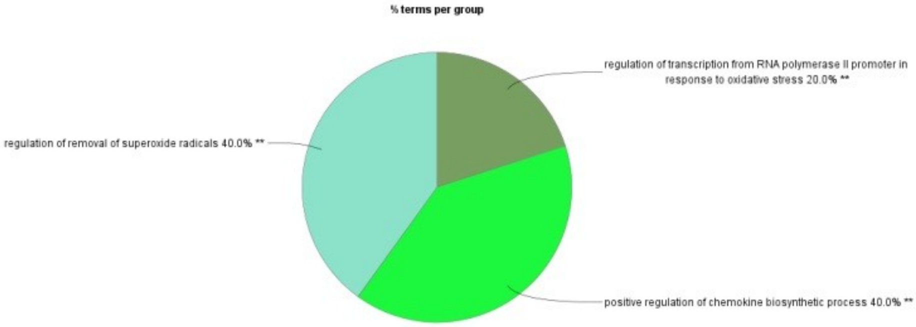

④ Luteolin

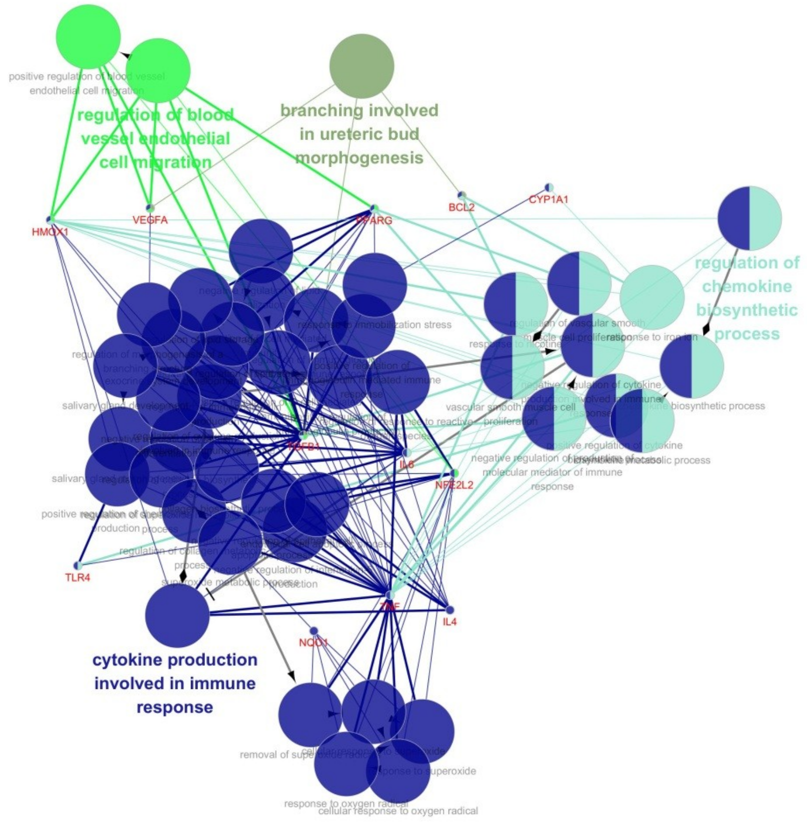
regulation of blood vessel endothelial cell migration
branching involved in ureteric bud morphogenesis
regulation of chemokine biosynthetic process
cytokine production involved in immune response
positive regulation of blood vessel endothelial cell migration
HMOX1
VEGFA
PPARG
BCL2
CYP1A1
TLR4
IL6
NFE2L2
IL4
NQO1
vascular smooth muscle cell proliferation
removal of superoxide radicals
response to superoxide
response to oxygen radical
cellular response to oxygen radical

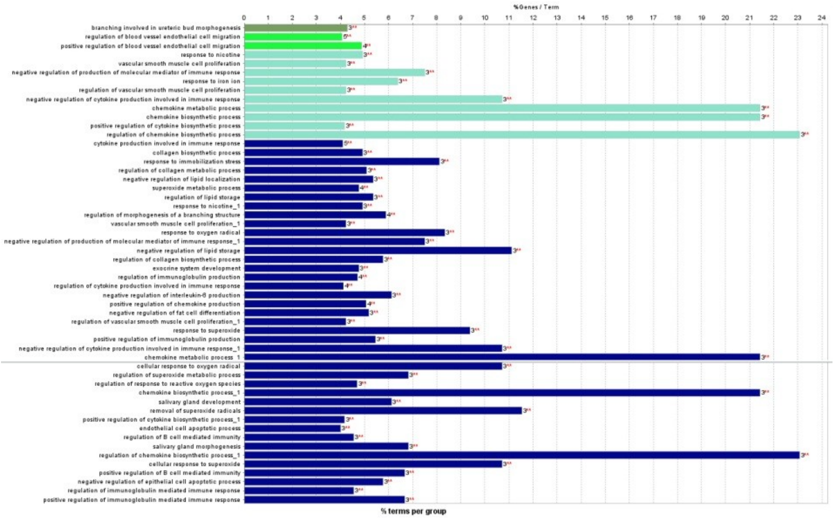
%Genes / Term
0 1 2 3 4 5 6 7 8 9 10 11 12 13 14 15 16 17 18 19 20 21 22 23 24
branching involved in ureteric bud morphogenesis
regulation of blood vessel endothelial cell migration
positive regulation of blood vessel endothelial cell migration
response to nicotine
vascular smooth muscle cell proliferation
negative regulation of production of molecular mediator of immune response
response to iron ion
regulation of vascular smooth muscle cell proliferation
negative regulation of cytokine production involved in immune response
chemokine metabolic process
chemokine biosynthetic process
positive regulation of cytokine biosynthetic process
regulation of chemokine biosynthetic process
cytokine production involved in immune response
collagen biosynthetic process
response to immobilization stress
regulation of collagen metabolic process
negative regulation of lipid localization
superoxide metabolic process
regulation of lipid storage
response to nicotine_1
regulation of morphogenesis of a branching structure
vascular smooth muscle cell proliferation_1
response to oxygen radical
negative regulation of production of molecular mediator of immune response_1
negative regulation of lipid storage
regulation of collagen biosynthetic process
exocrine system development
regulation of immunoglobulin production
regulation of cytokine production involved in immune response
negative regulation of interleukin-6 production
positive regulation of chemokine production
negative regulation of fat cell differentiation
regulation of vascular smooth muscle cell proliferation_1
response to superoxide
positive regulation of immunoglobulin production
negative regulation of cytokine production involved in immune response_1
chemokine metabolic process_1
cellular response to oxygen radical
regulation of superoxide metabolic process
regulation of response to reactive oxygen species
chemokine biosynthetic process_1
salivary gland development
removal of superoxide radicals
positive regulation of cytokine biosynthetic process_1
endothelial cell apoptotic process
regulation of B cell mediated immunity
salivary gland morphogenesis
regulation of chemokine biosynthetic process_1
cellular response to superoxide
positive regulation of B cell mediated immunity
negative regulation of epithelial cell apoptotic process
regulation of immunoglobulin mediated immune response
positive regulation of immunoglobulin mediated immune response
% terms per group

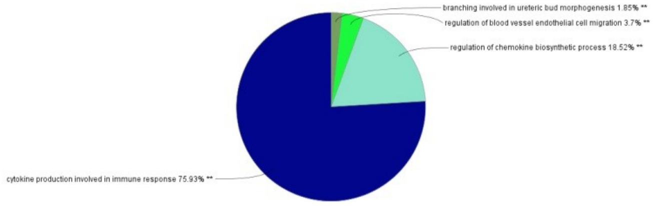
branching involved in ureteric bud morphogenesis 1.85% **
regulation of blood vessel endothelial cell migration 3.7% **
regulation of chemokine biosynthetic process 18.52% **
cytokine production involved in immune response 75.93% **

⑤ Chlorogenic acid

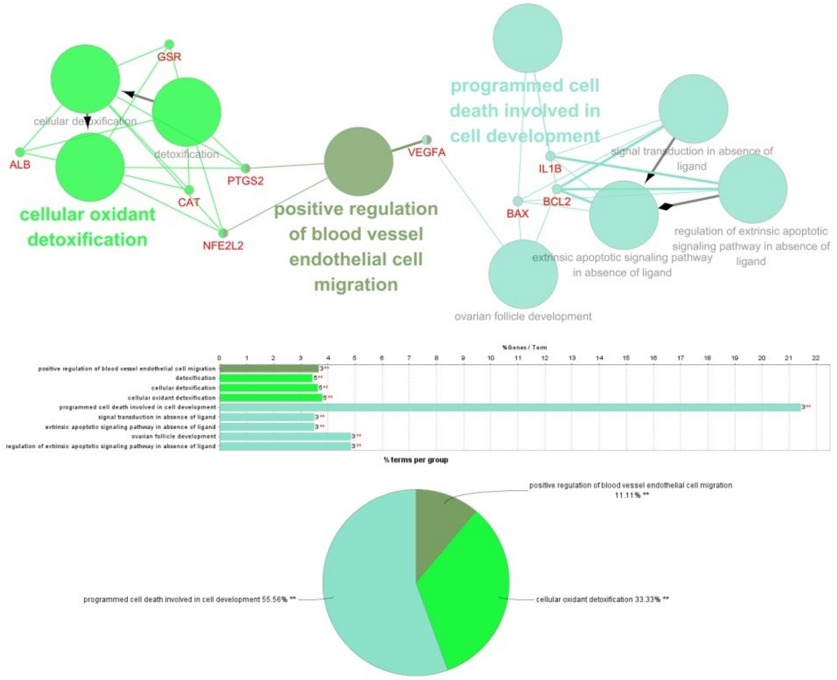


⑥ Emodin

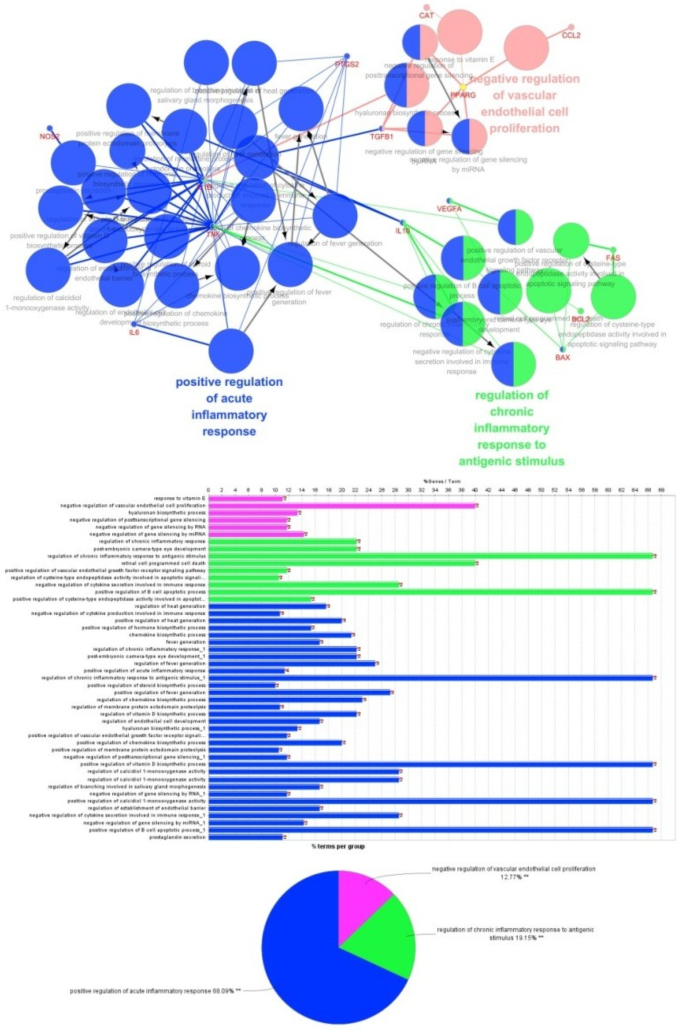

⑦ Betulin

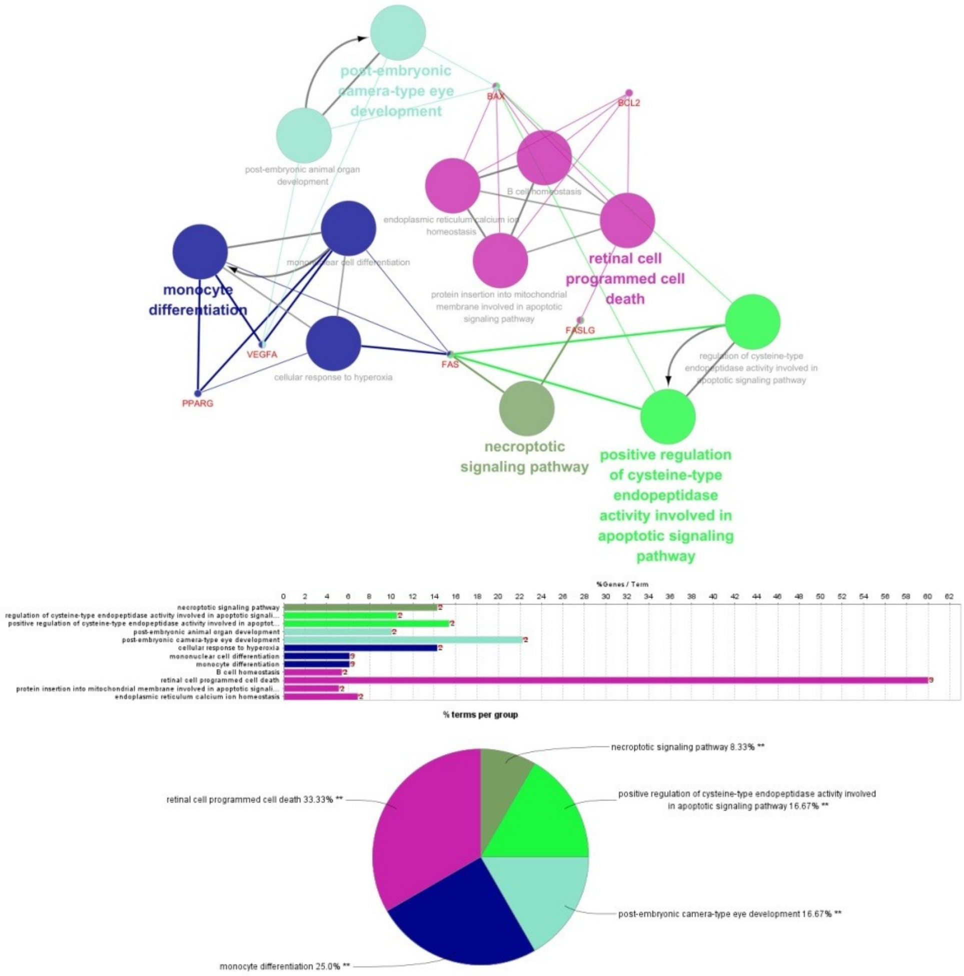


⑧ Apigenin

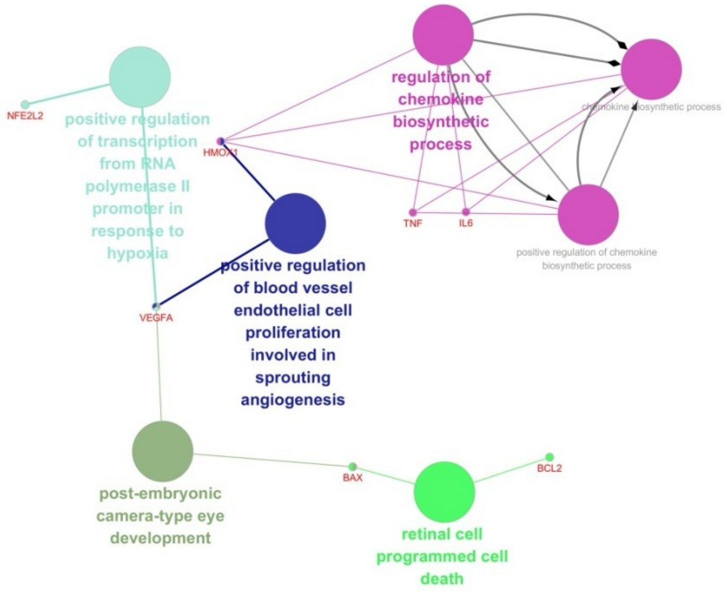

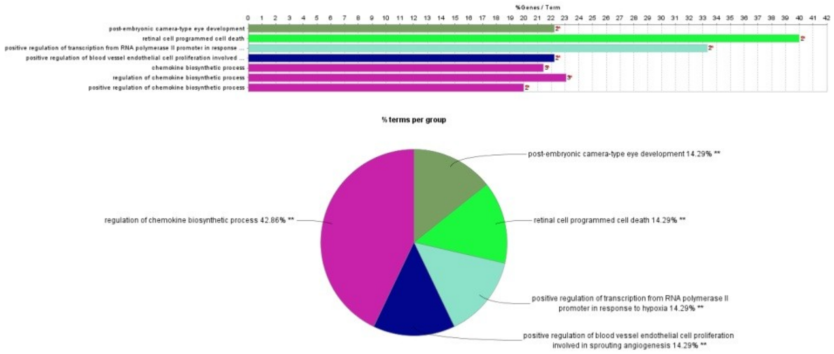


⑨ Hydroxycoumarin

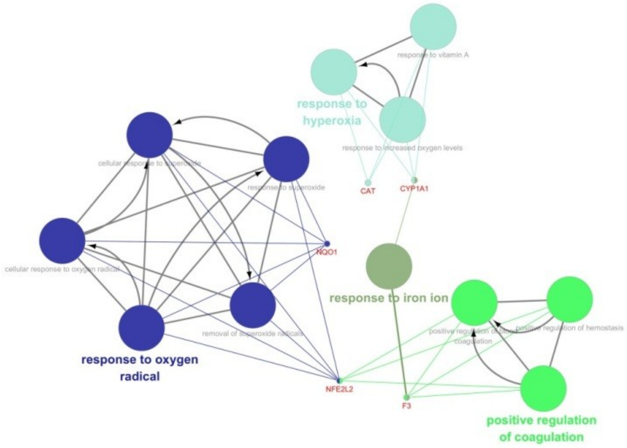


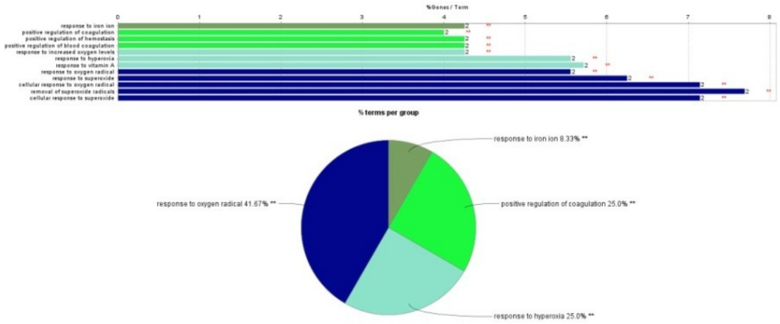


⑩ Oleanolic acid

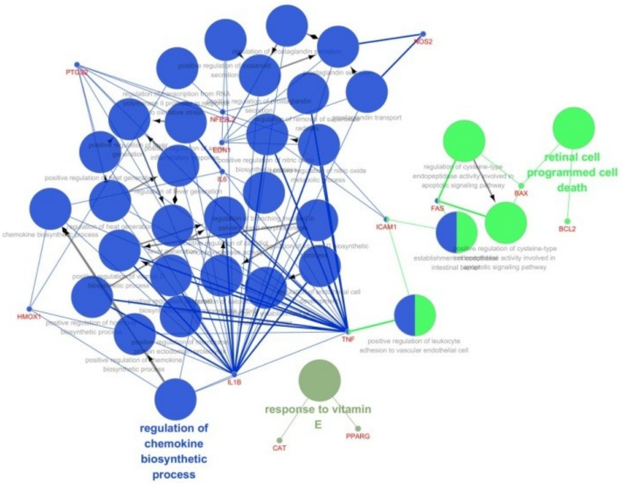

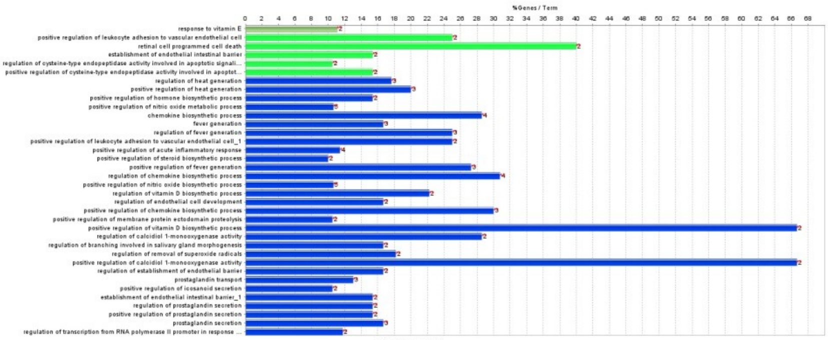


% terms per group

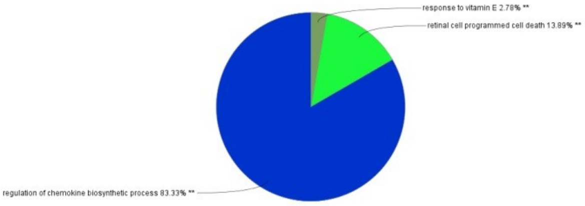

Supplement: Supplementary file 1 [file ijms-20-00543-s001.pdf]
